# In-hospital outcomes predictors and trends of redo sternotomy aortic root replacements: insights from a UK registry analysis

**DOI:** 10.3389/fcvm.2023.1295968

**Published:** 2024-01-08

**Authors:** Daniel P. Fudulu, Tim Dong, Rahul Kota, Shubhra Sinha, Jeremy Chan, Cha Rajakaruna, Arnaldo Dimagli, Gianni D. Angelini, Eltayeb Mohamed Ahmed

**Affiliations:** ^1^ Department of Cardiac Surgery, Bristol Heart Institute, University of Bristol, Bristol, United Kingdom; ^2^ Bristol Medical School, University of Bristol, Bristol, United Kingdom

**Keywords:** redo sternotomy, aortic root, root surgery, heart surgery, root replacement

## Abstract

**Background:**

Redo sternotomy aortic root surgery is technically demanding, and the evidence on outcomes is mostly from retrospective, small sample, single-centre studies. We report the trend, early clinical results and outcome predictors of redo aortic root replacement over 20 years in the United Kingdom.

**Methods:**

We retrospectively analysed collected data from the UK National Adult Cardiac Surgery Audit (NACSA) on all redo sternotomy aortic root replacements performed between 30th January 1998 and 19th March 2019. We analysed trends in the volume of operations, characteristics of hospital survivors vs. non-survivors, and predictors of in-hospital outcomes.

**Results:**

During the study period, 1,107 redo sternotomy aortic root replacements were performed (median age 59, 26% of patients were females). Eighty-four per cent of cases (*N* = 931) underwent a composite root replacement, 11% (*N* = 119) had homograft root replacement and valve-sparing root replacement was performed in 5.1% (*N* = 57) of cases. There was a steady increase in the volume of redo sternotomy root replacements beyond 2006, from an annual volume of 22 procedures in 2006 to 106 procedures in 2017. Hospital mortality was 17% (*n* = 192), postoperative stroke or TIA occurred in 5.2% (*n* = 58), and postoperative dialysis was required in 11% (*n* = 109) of patients. Return to the theatre for bleeding/tamponade was required in 9% (*n* = 102) and median in-hospital stay was 9 days. Age >59 (OR: 2.99, CI: 1.92–4.65, *P* < 0.001), recent myocardial infarction (OR: 6.42, CI: 2.24–18.41, *P* = 0.001) were associated with increased in-hospital mortality. Emergency surgery (OR: 3.95, 2.27–6.86, *P* < 0.001), surgery for endocarditis (OR: 2.05, CI: 1.26–3.33, *P* = 0.001), salvage coronary artery bypass grafting (OR: 2.20, CI: 1.37–3.54, *P* < 0.001), arch surgery (OR: 2.47, CI: 1.30–3.61, *P* = 0.018) and aortic cross-clamp longer than 169 min (OR: 2.17, CI: 1.00–1.01, *P* = 0.003) were associated with increased risk of mortality. We found no effect of the centre or surgeon volume on mortality (*P* > 0.05).

**Conclusions:**

Redo sternotomy aortic root replacement still carries significant morbidity and mortality and is sporadically performed across surgeons and centres in the UK.

## Introduction

In 1968 Bentall and De Bono described their technique of aortic root replacement in a 33-year-old patient (1). Over the years, the technique has evolved with a series of modifications (2), including replacing the aortic root while preserving the aortic valve (3). Aortic root replacement is routinely performed with a very low mortality (4). However, it is recognized that redo surgery on the aortic root is a strong predictor of postoperative mortality (4). Redo sternotomy aortic root replacement is often indicated in the setting of previous aortic valve surgery, aortic dissection, or infective endocarditis (5, 6). These indications have their inherent challenges that are compounded by the adversities caused by pericardial adhesions, difficulties in obtaining adequate exposure of the aortic root, mobilization of the coronary button, and the fragility of the native aortic tissues (7).

Furthermore, there is an ongoing debate over the prosthesis type to be used in these surgeries (8). The reported series of redo aortic root replacements in the literature are mostly retrospective, small sample size single-centre. Here we report the trend, early clinical results and outcome predictors of redo sternotomy aortic root replacement over 20 years in the United Kingdom.

## Patients and methods

### Study design and setting

We retrospectively analyzed collected data from the UK National Adult Cardiac Surgery Audit (NACSA), obtained from the National Institute of Cardiovascular Outcomes Research (NICOR) central cardiac database. The definitions of the database variables used for this study are found at https://www.nicor.org.uk/national-cardiac-audit-programme/adult-cardiac-surgery-surgery-audit/. The NACSA registry prospectively collects demographic, as well as pre-, peri-, and postoperative clinical data for all significant adult cardiac surgery procedures performed in the UK. Its central role is benchmarking surgical practice. The flow of the data from data input to analysis has been previously described. The data are entered locally and validated at the unit level by database managers before uploading through a web portal to NICOR. Further validation is performed according to logical rules, and missing data reports are generated for primary variables (e.g., EuroSCORE2 risk factors, patient identifiers and outcome data). The data are then forwarded to an academic healthcare informatics department for data cleaning. Duplicate records are removed, transcriptional discrepancies are re-coded, and clinical and temporal conflicts are resolved. Missing data are determined during the validation stages of the data transfer from individual centres. Missing and conflicting data for in-hospital mortality status are backfilled and validated via record linkage to the Office for National Statistics (ONS) census database. Missing data is reported in the baseline and outcome characteristics tables in the results section. We have treated missing data by exclusion from analysis.

### Patients

Patients who had redo sternotomy and replacement of the aortic root between 31 January 1998 and March 2019 were included in the study.

### Outcomes

The primary outcome was mortality. Secondary outcomes included neurological injury (composite outcome of reversible neurological deficit and permanent neurological deficit), postop renal impairment requiring dialysis, return to theatre for bleeding or cardiac tamponade, and hospital length of stay.

### Statistical methods

Categorical variables were summarised as counts and percentages and compared using a Pearson's Chi-squared test or Fisher's exact test. A Shapiro–Wilks test was used to assess the normality of the distribution of continuous data. Our continuous data were non-normally distributed, summarised as a median with interquartile range (IQR), and analysed using the Wilcoxon rank sum test. The *P* values were adjusted for multiple testing using the Bonferroni method.

We used a generalized linear mixed-effects model to assess the effect of several patient and procedural variables in patients undergoing aortic root replacement on the binary outcome of mortality. We performed Youden's J index analysis to select the optimal predicted probability cut-off for the aortic cross-clamp time, age, and preoperative creatine and these were found to be 169 min, age (>59 years) and preoperative creatine (Creatine >125 µmol/L). We imputed these variables as categorical in the model. Analysis was conducted in R Version 1.4.1106, packages: gtsummary, lme4 and sjPlot, ggplot2, cutpoint, cars. The candidate variables we included in the model as *fixed effects* were the following *patient* variables: gender, age >59 years, neurological dysfunction, renal dysfunction (Creatinine >125 µmol/L), recent myocardial infarction, pulmonary disease, unstable angina (CCS 4), NYHA class 4, pulmonary hypertension, diabetes on insulin, smoking history, left ventricular impairment, peripheral vascular disease, preoperative atrial fibrillation; we included the following *procedural* variables: the urgency of the procedure (emergency or urgent), previous aortic procedure (involving aortic root, ascending aorta and arch surgery), previous surgery involving aortic valve replacement (includes composite root replacement, isolated valve replacement, Ross procedure and homograft replacement), surgery for endocarditis, unplanned coronary artery bypass grafting (CABG)—defined as CABG performed in patients with no pre-existing coronary artery disease, aortic cross-clamp time (min), arch surgery and use of deep hypothermic circulatory arrest, number of operations per surgeon or centre before index procedure and 4-year time epoch when the surgery was performed. We treated the consultant code and hospital centre as *random intercept* variables. The variables included in the model were tested for multicollinearity, and none was found. There was no patient or public involvement in the design of this retrospective database analysis.

## Results

### Trends in the volume of redo root replacements

**Figure 1:**
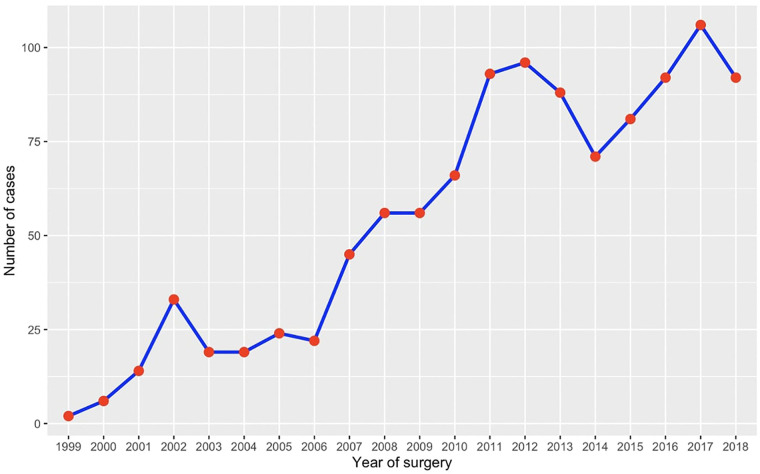
Trends in the volume of redo operations between January 1999 to March 2018.

The total volume of redo aortic root replacement over a 20-year period between January 01, 1999 and January 01, 2019 (*N* = 1,081) is depicted in Figure 1. For this trend analysis, we excluded operations performed between January 30, 1998 and January 2, 2019 to 19th of March 2019 to look at 20 full calendar years (*N* = 26). There was a steep and steady increase in the volume of redo procedures beyond 2006, from an annual volume of 22 procedures up to a peak of 106 procedures in 2017 (Figure 1). We found no effect of the 4-year time epoch on outcomes of surgery.

### Characteristics and outcomes in the whole cohort

Baseline characteristics for the overall group are divided by survivors and non-survivors (*N* = 1,107) and are depicted in Table 1. The median age was 59, 26% of cases (284) were females, and the median logistic Euroscore II was 6. The rest of the baseline characteristics are summarised in Table 1. The non-survivors were more likely to have had an emergency procedure and a higher Euroscore II (*P* < 0.001). Non-survivors had a higher prevalence of advanced age (*P* < 0.001), neurological dysfunction (*P* < 0.005), renal dysfunction (*P* < 0.001) and myocardial infarctions (*P* < 0.011) preoperatively. Twenty-seven per cent of patients (*N* = 296) had previous aortic procedures (ascending and aortic arch surgery), 66% of cases had previous valve surgery (aortic, mitral or tricuspid), and 6.8% had unclassified procedures. Thirty-two per cent of cases (*N* = 349) had previous aortic valve replacement (isolated or as part of a previous composite root replacement). The root pathology operated on is depicted in Table 1. Most of the patients underwent redo root replacements for aneurysmal dilatation (39%, *N* = 436), followed by intramural haematoma in 7.6% of cases (*N* = 84) and acute dissection (*N* = 42, 3.8% of cases). Missing data for root pathology operated on was 35% (*N* = 384). Redo root replacement for endocarditis was performed in 26% of cases. The majority of the redo roots replacements were composite root replacements (Bentall-De Bono operation) in 84% of cases (*N* = 931) performed with a mechanical valve in 39% of cases (*N* = 427) and a tissue valve in 29% of cases (*N* = 318). Missing data on the type of valve was 21% (*N* = 288). The rest of the procedures were homograft root replacements (11%, *N* = 119) and valve-sparing root replacements (5.1%, *N* = 57). Unplanned CABG was performed in 10% of cases (*N* = 112). The median CPB time was 234 min, and the median cross-clamp time was 156 min. The rest of the procedural characteristics are summarised in Table 2.

**Table 1:** Baseline characteristics of survivors and non-survivors following redo root replacement.

	Overall, *N* = 1,107^a^	Survivors, *N* = 915^a^	Non-survivors, *N* = 192^a^	*P*-value^b,c^
Female	284 (26%)	228 (25%)	56 (29%)	0.9
Age (years)	59 (47, 69)	58 (46, 68)	64 (55, 72)	<0.001
Neurological dysfunction	67 (6.1%)	44 (4.8%)	23 (12%)	<0.005
Creatinine >200 µmol/L	63 (5.7%)	33 (3.6%)	30 (16%)	<0.001
Recent myocardial infarction	25 (2.3%)	13 (1.4%)	12 (6.2%)	<0.011
Pulmonary disease	121 (11%)	92 (10%)	29 (15%)	0.041
CCS 4	31 (2.8%)	17 (1.9%)	14 (7.3%)	<0.001
NYHA4	132 (12%)	90 (9.8%)	42 (22%)	<0.001
Pulmonary hypertension	20 (1.8%)	13 (1.4%)	7 (3.6%)	>0.9
Diabetes on insulin	13 (1.2%)	10 (1.1%)	3 (1.6%)	>0.9
Smoking history (active or ex-smoker)	596 (54%)	507 (55%)	89 (46%)	0.7
LVF function				>0.9
Poor (EF <30%)	11 (1.0%)	7 (0.8%)	4 (2.1%)	
Moderate (EF 30%–50%)	193 (17%)	156 (17%)	37 (19%)	
Peripheral vascular disease	169 (15%)	144 (16%)	25 (13%)	>0.9
Preoperative atrial fibrillation	123 (11%)	93 (10%)	30 (16%)	>0.9
Emergency procedure	142 (13%)	87 (9.5%)	55 (29%)	<0.001
Urgent procedure	359 (32%)	293 (32%)	66 (34%)	>0.9
Salvage procedure	19 (1.7%)	4 (0.4%)	15 (7.8%)	<0.001
Euro score 2	6 (3, 13)	5 (3, 11)	13 (6, 26)	<0.001
Previous aortic procedures (ascending and arch)	296 (27%)	236 (26%)	60 (31%)	>0.9
Previous valve procedures (any valve repair or replacement surgery including mitral, aortic or tricuspid, etc.)	731 (66%)	600 (66%)	131 (68%)	>0.9
Previous other procedures (undefined)	75 (6.8%)	64 (7.0%)	11 (5.7%)	>0.9
Previous aortic valve replacement ± Root replacement	349 (32%)	276 (30%)	73 (38%)	>0.9
Aortic root pathology				
Acute dissection	42 (3.8%)	26 (2.8%)	16 (8.3%)	<0.009
Aneurysm	436 (39%)	397 (43%)	39 (20%)	<0.001
Chronic dissection	41 (3.7%)	39 (4.3%)	2 (1.0%)	>0.9
Iatrogenic dissection	18 (1.6%)	16 (1.7%)	2 (1.0%)	>0.9
Intramural haematoma	84 (7.6%)	71 (7.8%)	13 (6.8%)	>0.9
Penetrating atheromatous ulcer	16 (1.4%)	10 (1.1%)	6 (3.1%)	>0.9
Pseudoaneurysm	32 (2.9%)	26 (2.8%)	6 (3.1%)	>0.9
Trauma	54 (4.9%)	38 (4.2%)	16 (8.3%)	0.5
Missing data	384 (35%)	292 (32%)	92 (48%)	<0.001
Endocarditis	290 (26%)	207 (23%)	83 (43%)	<0.001

CCS, Canadian Cardiovascular Society; NYHA, New York Heart Association; LVF, left ventricular function; EF, ejection fraction.

^a^ *n* (%); Median (IQR).

^b^ Fisher's exact test; Wilcoxon rank sum test; Pearson's Chi-squared test.

^c^ Bonferroni correction for multiple testing.

**Table 2:** Procedural characteristics of survivors and non-survivors of redo root replacements.

Characteristic	Overall, *N* = 1,107^a^	Survivors, *N* = 915^a^	Nonsurvivors, *N* = 192^a^	*P*-value^b,c^
Type of root replacement				
Bentall	931 (84%)	778 (85%)	153 (80%)	>0.9
Homograft	119 (11%)	85 (9.3%)	34 (18%)	0.009
Valve sparing	57 (5.1%)	52 (5.7%)	5 (2.6%)	>0.9
Salvage CABG	112 (10%)	69 (7.5%)	43 (22%)	<0.001
Type of valve used for root prosthesis				
Biological	318 (29%)	240 (26%)	78 (41%)	<0.001
Mechanical	427 (39%)	373 (41%)	54 (28%)	0.015
Homograft	91 (8.2%)	68 (7.4%)	23 (12%)	0.5
Missing data	228 (21%)	194 (21%)	34 (18%)	>0.9
Aortic Arch Surgery	118 (11%)	92 (10%)	26 (14%)	>0.9
Descending Thoracic Aorta Surgery	33 (3.0%)	31 (3.4%)	2 (1.0%)	>0.9
CPB time (min)	234 (175, 320)	218 (170, 291)	350 (244, 444)	<0.001
Missing data	52	35	17	
Aortic Cross Clamp time (min)	156 (123, 203)	151 (121, 195)	193 (144, 248)	<0.001
Missing data	76	43	33	
Use of circulatory arrest	113 (10%)	81 (8.9%)	32 (17%)	0.016

^a^ *n* (%); Median (IQR).

^b^ Pearson's Chi-squared test; Wilcoxon rank sum test.

^c^ Bonferroni correction for multiple testing.

Hospital mortality was 17% (*n* = 192), postoperative stroke or TIA occurred 5.2% (*n* = 58), and postoperative dialysis was needed in 11% (*n* = 109) of cases. Re-exploration for bleeding or tamponade was required in 9% (*n* = 102) of patients. The median length of hospital stay was 11 days (IQR: 7,22) (Table 3).

**Table 3:** Crude outcomes of redo root replacements.

Characteristic	*N* = 1,107**^a^**
Mortality	192 (17%)
Postop TIA/CVA	58 (5.2%)
Postop renal dialysis	109 (11%)
Missing data	105
Return to theatre for bleeding/tamponade	102 (9.2%)
Length of stay (days)	12 (7, 22)
Missing data	8

^a^ *n* (%); Median (IQR).

### Patient characteristics of survivors and non-survivors

Non-survivors, in comparison with survivors, were older (64 vs. 58 years, *P* < 0.001) and had a higher median EuroScore II (13% vs. 5%, *P* < 0.001). They also had more preoperative neurological dysfunction (12% vs. 4.8%, *P* < 0.005), renal dysfunction (16% vs. 3.6%, *P* < 0.001), recent myocardial infarction (6.2% vs. 1.4%, *P* < 0.011), pulmonary disease (15% vs. 10%, *P* = 0.041), CCS class 4 symptoms (7.3% vs. 1.9%, *P* < 0.001) and NYHA class 4 symptoms (22% vs. 9.8%, *P* < 0.001). Among non-survivors, there were more patients undergoing emergency (29% vs. 9.5%, *P* < 0.001) and salvage procedures (7.8% vs. 0.4%, *P* < 0.001), reoperation for acute aortic dissection (8.3% vs. 2.8%, *P* < 0.009), and surgery for infective endocarditis (43% vs. 23%, *P* < 0.001). Moreover, there were fewer patients with aneurysms (20% vs. 43%, *P* < 0.001) (Table 1).

### Procedural characteristics of survivors and non-survivors

Non-survivors had more frequent use of homografts (18% vs. 9.3%, *P* = 0.009) and twice more associated salvage CABG procedures (22% vs. 7.5%, *P* < 0.001). The use of a biological valve for the composite root replacement was more common amongst non-survivors (41% vs. 26%, *P* < 0.001), while the use of a mechanical valve was less common in this group (28% vs. 41%, *P* = 0.015). The median cardiopulmonary bypass and aortic cross-clamp times were longer in non-survivors (350 min vs. 218 min, *P* < 0.001 and 193 vs. 151 min, *P* < 0.001, respectively). Circulatory arrest was more common in the non-survivor group (17% vs. 8.9%, *P* = 0.016) (Table 2).

In Table 4, we have presented the baseline and procedural characteristics stratified by any redo procedure or previous aortic root (“true” re-do root replacement) or aortic valve procedure. Re-do root surgery following previous root procedure or aortic valve surgery was more often performed urgently (40% vs. 29%, *P* = 0.005), associated with a higher median EuroScore 2 (9 vs. 5, *P* < 0.001), and were more commonly performed for endocarditis (52% vs. 15%, *P* < 0.001).

**Table 4:** Characteristics and outcomes stratified by root pathology (any re-do versus re-do root or aortic valve replacement).

Characteristic	Any redo, *N* = 758^a^	Re-do root (true redo) or aortic valve, *N* = 349^a^	*P*-value^b^^,^^c^
Female	184 (24%)	100 (29%)	>0.9
Age (years)	58 (46, 68)	61 (49, 70)	0.5
Neurological dysfunction	49 (6.5%)	18 (5.2%)	>0.9
Creatinine > 200 µmol/L	32 (4.2%)	31 (8.9%)	0.079
Recent myocardial infarction	16 (2.1%)	9 (2.6%)	>0.9
Pulmonary disease	73 (9.6%)	48 (14%)	>0.9
CCS 4	17 (2.2%)	14 (4.0%)	>0.9
NYHA4	85 (11%)	47 (13%)	>0.9
Pulmonary hypertension	13 (1.7%)	7 (2.0%)	>0.9
Diabetes on insulin	4 (0.5%)	9 (2.6%)	0.2
Smoking history (active or ex-smoker)	336 (37%)	93 (48%)	0.076
LVF function			
Poor (EF <30%)	211 (28%)	86 (25%)	>0.9
Moderate (EF 30%–50%)	128 (17%)	65 (19%)	0.5
Peripheral vascular disease	130 (17%)	39 (11%)	0.4
Preoperative atrial fibrillation	96 (13%)	27 (7.7%)	0.6
Emergency procedure	87 (11%)	55 (16%)	>0.9
Urgent procedure	218 (29%)	141 (40%)	0.005
Salvage procedure	10 (1.3%)	9 (2.6%)	>0.9
Euro score II	5 (3, 11)	9 (5, 18)	<0.001
Unknown	2	0	
Aortic root pathology			
Acute dissection	31 (4.1%)	11 (3.2%)	>0.9
Aneurysm	318 (42%)	118 (34%)	0.4
Chronic dissection	35 (4.6%)	6 (1.7%)	0.7
Iatrogenic	17 (2.2%)	1 (0.3%)	0.7
Intramural haematoma	70 (9.2%)	14 (4.0%)	0.10
Penetrating atheromatous ulcer	10 (1.3%)	6 (1.7%)	>0.9
Pseudoaneurysm	16 (2.1%)	16 (4.6%)	>0.9
Trauma	30 (4.0%)	24 (6.9%)	>0.9
Missing data aortic root pathology	231 (30%)	153 (44%)	<0.001
Endocarditis	110 (15%)	180 (52%)	<0.001
Type of root replacement			
Bentall	61 (8.0%)	58 (17%)	<0.001
Homograft	648 (85%)	283 (81%)	>0.9
Valve sparing	49 (6.5%)	8 (2.3%)	0.2
Salvage CABG	118 (16%)	66 (19%)	>0.9
Type of valve used for root prosthesis			
Biological	173 (23%)	145 (42%)	<0.001
Mechanical	34 (4.5%)	57 (16%)	<0.001
Homograft	290 (38%)	137 (39%)	>0.9
Missing data	261 (34%)	10 (2.9%)	<0.001
Aortic arch surgery	99 (13%)	19 (5.4%)	0.006
Descending thoracic aorta surgery	29 (3.8%)	4 (1.1%)	0.6
CPB time (min)	236 (175, 323)	230 (175, 308)	>0.9
Missing data	27	25	
Aortic Cross Clamp time (min)	152 (123, 202)	162 (125, 209)	>0.9
Missing data	49	27	
Use of circulatory arrest	88 (12%)	25 (7.2%)	>0.9
Mortality	119 (16%)	73 (21%)	>0.9
Postop TIA/CVA	41 (5.4%)	17 (4.9%)	>0.9
Postop Renal Dialysis	75 (11%)	34 (11%)	>0.9
Missing data	77	28	
Return to theatre for bleeding/tamponade	73 (9.6%)	29 (8.3%)	>0.9
Length of stay (days)	11 (7, 20)	13 (7, 26)	>0.9
Missing data	8	0	

^a^ *n* (%); Median (IQR).

^b^ Fisher’s exact test; Wilcoxon rank sum test; Pearson’s Chi-squared test.

^c^ Bonferroni correction for multiple testing.

### Significant predictors of adverse outcomes

Age >59 years (OR: 2.99, CI: 1.92–4.65, *P* < 0.001), and recent MI (OR: 6.42, CI: 2.24–18.41, *P* < 0.001) were preoperative characteristics associated with increased in-hospital mortality. In terms of procedural predictors, emergency surgery (OR: 3.95, CI: 2.27–6.86, *P* < 0.001), surgery for endocarditis (OR: 2.20, CI: 1.37–3.54, *P* = 0.001), salvage CABG (OR: 2.63, CI: 1.58–4.38, *P* < 0.001), arch surgery (OR: 2.47, CI: 1.17–5.23, *P* = 0.018) and aortic cross-clamp > 169 min (OR: 2.17, CI: 1.30–3.61, *P* = 0.003) were associated with an increased risk of mortality (Table 5 and Figure 1).

**Table 5:** Effect estimates of various patient and procedural predictors on mortality using a generalized linear mixed model.

	Mortality		
*Predictors*	Odds ratios	CI	*P*
Female sex	1.56	0.99–2.47	0.055
Age >59 years	2.99	1.92–4.65	<0.001
Neurological dysfunction	1.74	0.83–3.67	0.143
Creatinine > 125 µmol/L	1.78	0.89–3.56	0.105
Recent MI	6.42	2.24–18.41	0.001
Pulmonary disease	1.29	0.69–2.41	0.419
CCS 4	2.28	0.81–6.39	0.118
NYHA IV	1.34	0.74–2.41	0.334
Pulmonary HTN	1.97	0.52–7.36	0.316
Diabetes on insulin	1.47	0.27–7.97	0.652
Poor LV function (EF <30%)	1.11	0.69–1.77	0.675
Peripheral vascular disease	0.75	0.41–1.36	0.342
Emergency surgery	3.95	2.27–6.86	<0.001
Previous aortic valve or composite root	1.44	0.89–2.35	0.140
Previous Ascending Aorta/Arch Surgery	1.42	0.86–2.33	0.166
Endocarditis	2.20	1.37–3.54	0.001
Homograft replacement	1.13	0.60–2.16	0.701
Valve Sparing procedure	0.64	0.19–2.21	0.480
Salvage CABG	2.63	1.58–4.38	<0.001
Aortic cross-clamp time >169 min	2.17	1.30–3.61	0.003
Arch surgery	2.47	1.17–5.23	0.018
Use of deep hypothermic circulatory arrest	1.88	0.96–3.69	0.068
Reference—low volume centre			
Medium volume centre	1.37	0.76–2.45	0.292
High volume centre	0.93	0.49–1.77	0.834
Reference—low volume surgeon			
Medium volume consultant	0.95	0.56–1.60	0.833
High volume consultant	1.00	0.52–1.92	0.991
Reference 1998–2004			
2005–2009	1.43	0.56–3.64	0.456
2010–2014	1.21	0.47–3.11	0.697
2015–2019	1.01	0.37–2.79	0.978
Random effects			
*σ*^2^	3.29		
*τ*_00__Consultant_	0.32		
τ_00__HospCode_	0.29		
ICC	0.16		
*N*_Consultant_	286		
*N*_HospCode_	42		
Observations	1,029		
Marginal *R*^2^/Conditional *R*^2^	0.327/0.432		

### Effect of hospital and surgeon volume on outcomes

**Figure 2:**
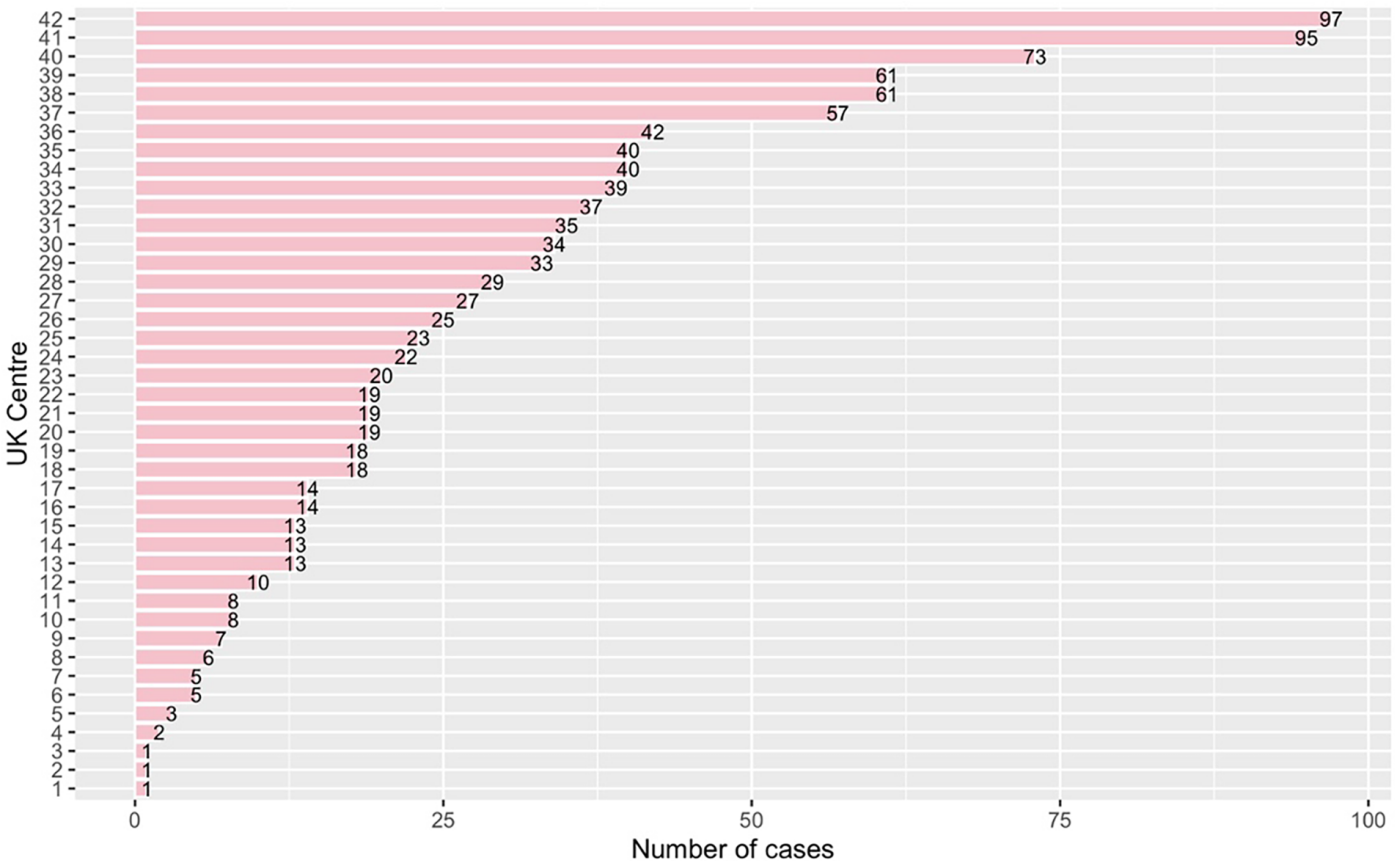
Number of cases per UK centre within the study period.

A total of 304 surgeons performed re-do sternotomy root replacements during the study period. The number of cases per consultant ranged from 1 to 49, vmedian number of cases per consultant was 2 (IQR: 3–13). A total of 42 centres performed re-do sternotomy root replacements ranging from 1 case per centre to 97 cases per centre during the study period, with the median number of cases per centre being 19 (IQR: 8.5–36.5) (Figure 2). We analysed the effect of the number of cases per surgeon and centre before each procedure to consider the accumulating experience throughout the study period. The number of cases before index procedure per surgeon was classified into terciles, and surgeons were defined as low volume (</=4 cases before index procedure), medium volume (>4 cases and </=10 cases before index procedure) and high volume (>10 cases before index procedure). According to the above definition, 481 cases were operated by low-volume surgeons, 287 by medium volume and 339 by high-volume surgeons. Similarly, we defined units as low-volume units (</= 10 cases), medium-volume (>10 cases and </=26 cases) and high-volume (>26 cases). This resulted in 387 cases being operated on by low-volume centres, 352 by medium-volume centres and 368 by high-volume centres. In Table 6, we have stratified the baseline and procedural characteristics by centre volume. There was no difference in baseline characteristics (all *P* > 0.9).

**Table 6:** Characteristics and outcomes of patients stratified by centre volume of procedures.

Characteristic	Low volume unit, *N *=* *387^a^	Medium volume unit, *N* = 352^a^	High volume unit, *N* = 368^a^	Low vs. medium *P*-value^b,c^	Low vs. high *P*-value^b,c^	Medium vs. high *P*-value^b,c^
Female	98 (25%)	86 (24%)	100 (27%)	>0.9	>0.9	>0.9
Age (years)	59 (47, 69)	60 (47, 68)	58 (46, 69)	>0.9	>0.9	>0.9
Neurological dysfunction	27 (7.0%)	15 (4.3%)	25 (6.8%)	>0.9	>0.9	>0.9
Creatinine > 200 µmol/L	20 (5.2%)	20 (5.7%)	23 (6.3%)	>0.9	>0.9	>0.9
Recent myocardial infarction	12 (3.1%)	4 (1.1%)	9 (2.4%)	>0.9	>0.9	>0.9
Pulmonary disease	40 (10%)	39 (11%)	42 (11%)	>0.9	>0.9	>0.9
CCS 4	16 (4.1%)	10 (2.8%)	5 (1.4%)	>0.9	>0.9	>0.9
NYHA4	41 (11%)	42 (12%)	49 (13%)	>0.9	>0.9	>0.9
Pulmonary hypertension	4 (1.0%)	8 (2.3%)	8 (2.2%)	>0.9	>0.9	>0.9
Diabetes on insulin	3 (0.8%)	6 (1.7%)	4 (1.1%)	>0.9	>0.9	>0.9
Smoking history (active or ex-smoker)	143 (37%)	134 (38%)	152 (41%)			
LVF function				>0.9	>0.9	>0.9
Poor (EF <30%)	6 (1.6%)	4 (1.1%)	1 (0.3%)			
Moderate (EF 30%–50%)	58 (15%)	70 (20%)	65 (18%)			
Peripheral vascular disease	54 (14%)	58 (16%)	57 (15%)	>0.9	>0.9	>0.9
Preoperative atrial fibrillation	48 (12%)	37 (11%)	38 (10%)	>0.9	>0.9	>0.9
Emergency procedure	51 (13%)	41 (12%)	50 (14%)	>0.9	>0.9	>0.9
Urgent procedure	120 (31%)	117 (33%)	122 (33%)	>0.9	>0.9	>0.9
Salvage procedure	7 (1.8%)	10 (2.8%)	2 (0.5%)	>0.9	>0.9	0.7
Euro score 2	7 (3, 13)	6 (3, 14)	5 (3, 11)	>0.9	>0.9	>0.9
Unknown	0	2	0			
Previous other procedures (undefined)	98 (25%)	89 (25%)	109 (30%)	>0.9	>0.9	>0.9
Previous aortic valve replacement ± Root replacement	249 (64%)	241 (68%)	241 (65%)	>0.9	>0.9	>0.9
Previous other procedures (undefined)	23 (5.9%)	29 (8.2%)	23 (6.3%)	>0.9	>0.9	>0.9
Aortic root pathology						
Acute dissection	25 (6.5%)	9 (2.6%)	8 (2.2%)	0.5	0.5	>0.9
Aneurysm	179 (46%)	146 (41%)	111 (30%)	>0.9	>0.9	0.065
Chronic dissection	15 (3.9%)	11 (3.1%)	15 (4.1%)	>0.9	>0.9	>0.9
Iatrogenic dissection	2 (0.5%)	6 (1.7%)	10 (2.7%)	>0.9	>0.9	>0.9
Intramural haematoma	8 (2.1%)	16 (4.5%)	60 (16%)	>0.9	>0.9	<0.001
Penetrating atheromatous ulcer	7 (1.8%)	5 (1.4%)	4 (1.1%)	>0.9	>0.9	>0.9
Pseudoaneurysm	11 (2.8%)	10 (2.8%)	11 (3.0%)	>0.9	>0.9	>0.9
Trauma	11 (2.8%)	15 (4.3%)	28 (7.6%)	>0.9	>0.9	>0.9
Missing data	129 (33%)	134 (38%)	121 (33%)	>0.9	>0.9	>0.9
Endocarditis	106 (27%)	90 (26%)	94 (26%)	>0.9	>0.9	>0.9
Type of root replacement						
Bentall	46 (12%)	37 (11%)	36 (9.8%)	>0.9	>0.9	>0.9
Homograft	302 (78%)	309 (88%)	320 (87%)	0.002	0.061	>0.9
Valve sparing	39 (10%)	6 (1.7%)	12 (3.3%)	<0.001	0.009	>0.9
Salvage CABG	63 (16%)	50 (14%)	71 (19%)	>0.9	>0.9	>0.9
Type of valve used for root prosthesis						
Biological	102 (26%)	99 (28%)	109 (30%)	>0.9	>0.9	>0.9
Mechanical	32 (8.3%)	28 (8.0%)	31 (8.4%)	>0.9	>0.9	>0.9
Homograft	143 (37%)	147 (42%)	131 (36%)	>0.9	>0.9	>0.9
Missing data	71 (18%)	72 (20%)	85 (23%)	>0.9	>0.9	>0.9
Aortic arch surgery	19 (4.9%)	22 (6.3%)	77 (21%)	>0.9	>0.9	<0.001
Descending thoracic aorta surgery	0 (0%)	2 (0.6%)	31 (8.4%)	>0.9	>0.9	<0.001
CPB time (min)	242 (179, 322)	222 (177, 309)	233 (168, 321)	>0.9	>0.9	>0.9
Missing data	17	18	17			
Aortic Cross Clamp time (min)	160 (126, 208)	155 (128, 206)	154 (118, 200)	>0.9	>0.9	>0.9
Missing data	31	24	21			
Use of circulatory arrest	43 (11%)	37 (11%)	33 (9.0%)	>0.9	>0.9	>0.9
Mortality	70 (18%)	64 (18%)	58 (16%)	>0.9	>0.9	>0.9
Postop TIA/CVA	16 (4.1%)	21 (6.0%)	15 (4.1%)	>0.9	>0.9	>0.9
Postop Renal Dialysis	30 (8.3%)	38 (12%)	41 (12%)	>0.9	>0.9	>0.9
Missing data	25	42	38			
Return to theatre for bleeding/tamponade	42 (11%)	35 (9.9%)	25 (6.8%)	>0.9	>0.9	>0.9
Length of stay (days)	11 (7, 19)	12 (7, 22)	12 (7, 22)	>0.9	>0.9	>0.9
Missing data	6	1	1			

^a^ *n* (%); Median (IQR).

^b^ Fisher’s exact test; Kruskal–Wallis rank sum test; Pearson’s Chi-squared test.

^c^ Bonferroni correction for multiple testing.

After adjusting the model for the rest of the covariates, we found no effect of medium volume centre (OR:1.37, CI: 0.76–2.45, *P* = 0.292) and high-volume centre (OR: 0.93, CI: 0.49–1.77, *P* = 0.834) on mortality. When the model was adjusted by volume of operations per surgeon, again, we found no effect of medium-volume surgeons (OR: 1.37, CI: 0.76–2.45, *P* = 0.292) and high-volume surgeons (OR: 1.00, CI: 0.20–1.34, 0.179) on mortality.

## Discussion

Redo aortic root replacement is technically demanding and often performed by surgeons with a special interest in aortic disease at experienced centres. The number of cases performed in each centre is generally small. One of the largest reported series in the literature by Urbanski (9), included 112 patients over 14 years. Therefore, the available literature is subject to publication and selection bias. Hence, there is a need to inform patients and clinicians from a large cohorts of patients. The National Adult Cardiac Surgery Audit (NACSA) collects prospective real-world data from all adult cardiac surgery centres in the United Kingdom, including over one thousand consecutive redo aortic root surgery patients. Therefore, it is well-suited to try to address the gap in evidence.

### Trends in the number of cases performed

The total number of redo aortic root replacements has increased over the last decade, which mirrors the increase in the number of first-time aortic root replacement surgeries (4). A similar trend was seen in the UK, with a steep and steady increase from an annual volume of 22 procedures in 2006 to a peak of 112 procedures in 2017.

Gaudino's (4) reported an increase in aortic root replacement together with a rise in the use of bioprosthesis and valve-sparing root replacement in a cohort with a mean age of 61 and 48 years of age, respectively. A UK-wide study showed a similar increase in the utilization of bioprosthesis; the same study reported freedom from structural valve degeneration or death of 30%–40% at 10 years (10). Furthermore, valve-sparing root replacement as a proportion of aortic root surgery tripled over the last decade (11). This increase in bioprosthesis and valve-sparing root replacement is a potential substrate for redo aortic root surgery. In a series of redo root replacements, 91% of patients had a previous root replacement or aortic valve replacement, 9% had previous valve-sparing root replacement, 22% had a previous homograft, and prosthetic valve dysfunction was the indication in over a quarter of patients (12). Patients who survive a type A aortic dissection repair are at risk of aortic root dilatation or pseudoaneurysm formation following an initial supracomissural repair or root replacement (13). Indeed, this cohort of patients represented over 80% of patients in Dossche series (14). Therefore, the surgical community should expect a steady increase in the volume of patients in need of reintervention on the aortic root.

### Effect of surgeon volume and centre on outcomes

Our analysis found no effect of surgeon and hospital centre on outcomes. Because re-do sternotomy root replacements are performed sporadically compared to other routine procedures, one explanation for the results is that volume of surgeries per surgeon/centre was not high enough to detect a signal in our analysis. Other work has also shown that due to standardisation in techniques and perioperative care in cardiac surgery, the impact of hospital and surgeon on outcomes is not that pronounced in cardiac surgery compared to other surgical specialities. Finally, other metrics apart from surgeon and volume can impact outcomes such as the phase of care mortality analysis or the failure to rescue (15, 16). Another hypothesis behind no effect of centre volume on outcomes is that the high-volume centres take on more complex root cases with *a priori* chance of worser outcomes, compared to low-volume centres. However, we found no differences in the baseline characteristics between low, medium or high-volume centres (Table 4).

### Mortality

The mortality in our NACSA cohort over 22 years was 17%. Higher than what was reported by other authors in the range of 3%–11% (9, 17). While it is plausible that the experience of the centre has a profound effect on the outcome, in one of the series, the mean age was 15 years lower than in the NACSA (17); the same group mortality increased from 3% to 7% in series reported a few years later, which may reflect a change in selection (18). Other series had a very small number of patients over two decades, which suggests an element of selection bias (5). Urbanski (9) reported a mortality of 11% in a cohort of a similar age to the NACSA, with a mortality of 16% at one year. Therefore, our study provides a real-world outlook from many units into the outcome of all-comers undergoing redo aortic root replacements.

### Predictors of adverse outcome

In our study, recent myocardial infarction (MI) was the strongest mortality risk predictor on multivariable analysis (OR: 6.42, CI: 2.24–18.41, *P* < 0.001); this was consistent with previous reports in the literature (12). Age was identified as a predictor for death in a series of 164 patients (17). We also found a cut-off of above 59 years at the time of surgery to be a predictor of postoperative mortality (OR: 2.99, CI: 1.92–4.65, *P* < 0.001).

Predictably, emergency surgery was associated with about four-fold increase in the risk of mortality (OR:3.95, CI 2.27–6.86, *P* < 0.001). In our experience, we find that previous aortic surgery increases the difficulty of redo aortic root interventions. However, previous aortic procedures were not associated with an increase in risk in our analysis. Furthermore, previous aortic valve replacement or composite root replacement did not significantly affect mortality. It is possible that redo root replacement in the context of a previous major aortic surgery is only performed in super-specialized centres.

Coronary artery bypass grafting may be indicated within the setting of redo surgery for native coronary artery disease. However, it is occasionally required to address right ventricular dysfunction, to deal with difficulties mobilizing or suturing the coronary buttons, or indeed damage to the coronary arteries during redo dissection. We identified salvage CABG as an independent predictor for mortality (OR: 2.63, CI: 1.58–4.38, *P* < 0.001). Kirsch (19) performed concomitant CABG in 16% of their redo aortic surgery series, unplanned CABG was the only independent predictor for mortality. The effect of emergency CABG was demonstrated by Keeling (20). In their series, the mortality of patients who required emergency CABG was 28%; conversely, patients who had CABG for coronary artery disease had a mortality of 6.3%, similar to isolated root replacement.

Infective endocarditis (IE) of prosthetic valve replacement is a serious complication; the rate of IE following aortic valve intervention is reported to be as high as 3% (21). Indeed, infective endocarditis was the indication for reintervention in 20% of cases in previous reports (22). In our study, redo root replacement for endocarditis was the indication for reintervention in around 26% of cases. Several techniques have been described to address the aortic root and annular destruction induced by IE, yet the intraoperative challenge is pronounced (9). In patients with IE of the prosthetic valve, mortality is four times higher than in non-IE patients (14, 23). In our work, the presence of IE resulted in a doubling of the risk of postoperative mortality (OR:2.20, CI: 1.37–3.54, *P* = 0.001). The increase in risk may be secondary to technical challenges or clinical instability of the patients that may not have been captured adequately in the data.

Cardiopulmonary bypass (CPB) during cardiac surgery initiates a systemic inflammatory response, resulting in a multiorgan injury. CPB duration and ischaemic time in cardiac surgery were associated with an increase in mortality (24, 25). The mean cross-clamp time in our study was 156 (IQ: 123, 203), 151 (IQ: 121, 195), and 193 (IQ: 144, 248) for the whole cohort, survivors and non-survivors, respectively. Using the Younden metric, we identified a cut-off point of 169 min beyond which mortality increases was identified. When adjusting in the model for the rest of the covariates, cross-clamp time was identified as an independent predictor for mortality (OR: 2.17, CI: 1.30–3.61, *P* = 0.003). In the Toronto General series, the reported mortality was 3%, and the average ischaemic time was 123 min with a cumulative CBP time of 163 min; similar results were reported in conjunction with a relatively shorter ischaemic time (17, 22).

Hypothermic circulatory arrest (HCA) with or without cerebral perfusion was more often used in non-survivors but was not an independent predictor for postoperative mortality; these findings are in line with other reports in the literature (26). It is possible that the variability in duration, adjuncts of cerebral protection used, and extensiveness of aortic reconstruction makes the evaluation of the impact of HCA in isolation not possible. However, the rate of arch reconstruction was similar in all groups. Therefore, it is likely that HCA was mostly used to perform an open distal aortic anastomosis or limited hemiarch reconstruction, which has been previously reported not to impact outcome (27).

In one study, a cryopreserved homograft was used in 95 redo aortic root replacements for IE; the authors reported a mortality of 18% for the whole cohort and freedom from reintervention of 95% at 17 years; the mortality in their cohort is similar to our study and the freedom from reintervention is certainly impressive (28). In the current study, more of the non-survivors had their aortic root replaced using a homograft (12% vs. 7.4%, p = 0.5); however, the use of homograft was not a predictor for increased in-hospital mortality.

### Strengths and limitations

This is an extensive series of redo aortic root replacement outcomes in a multicentre registry dataset that reflects real-world practice. The relatively large sample size allowed us to investigate multiple predictors of adverse outcomes for patients undergoing a high-risk procedure that is infrequently performed. As with any registry study, one limitation is the lack of data granularity. Specific to the current study is the lack of details around the previous procedure performed, mainly whether the previous procedure was a composite root, making the redo procedure a true redo root replacement, or isolated aortic valve replacement (29). Likely, this data is recorded as previous aortic valve replacement/explanation (*N* = 349) as part of the composite root replacement, homograft replacement or Ross procedure. However, we cannot separate the composite roots from previous aortic valve replacements in this group. Interestingly, the mortality for this subgroup was 21% (73 out of 249), slightly higher than the overall study mortality of 17%. However, this is much higher compared to the single centre study of “true” re-do root replacements by Malivandi et al. (12), which reports a mortality of 6.5% in a series of 46 patients and the study by Bavaria et al. that, reports a mortality of 5% on a sample size of 120 patients (27). Adjusting for this covariate was not associated with adverse outcomes (OR 1.44, 95% CI: 0.89–2.35, *P* = 0.140). This result is similar to a propensity-matched analysis by Patel et al. (30) of 638 patients who underwent any re-do roots vs. 184 patients who underwent true re-do root replacements which found no increase in mortality and morbidity with true-redo root replacement. Another limitation is the lack of long-term survival data.

## Conclusion

Redo sternotomy aortic root replacement still carries significant mortality and is sporadically performed across surgeons and centres. Our study from a nationwide multicentre registry dataset reflects real-world practice in the United Kingdom, which allowed us to investigate multiple predictors of adverse outcomes. This information will provide much-needed insight for patients and surgeons.

## Data Availability

The original contributions presented in the study are included in the article/Supplementary Material, further inquiries can be directed to the corresponding author.
